# Artificial intelligence, bias and clinical safety

**DOI:** 10.1136/bmjqs-2018-008370

**Published:** 2019-01-12

**Authors:** Robert Challen, Joshua Denny, Martin Pitt, Luke Gompels, Tom Edwards, Krasimira Tsaneva-Atanasova

**Affiliations:** 1 EPSRC Centre for Predictive Modelling in Healthcare, University of Exeter College of Engineering Mathematics and Physical Sciences, Exeter, UK; 2 Taunton and Somerset NHS Foundation Trust, Taunton, UK; 3 Departments of Biomedical Informatics and Medicine, Vanderbilt University Medical Center, Nashville, Tennessee, USA; 4 NIHR CLAHRC for the South West Peninsula, St Luke’s Campus, University of Exeter Medical School, Exeter, UK

**Keywords:** artificial intelligence, clinical safety, clinical decision support systems, machine learning

## Introduction

In medicine, artificial intelligence (AI) research is becoming increasingly focused on applying machine learning (ML) techniques to complex problems, and so allowing computers to make predictions from large amounts of patient data, by learning their own associations.^1^ Estimates of the impact of AI on the wider economy globally vary wildly, with a recent report suggesting a 14% effect on global gross domestic product by 2030, half of which coming from productivity improvements.^2^ These predictions create political appetite for the rapid development of the AI industry,^3^ and healthcare is a priority area where this technology has yet to be exploited.^2 3^ The digital health revolution described by Duggal *et al* 4 is already in full swing with the potential to ‘disrupt’ healthcare. Health AI research has demonstrated some impressive results,^5–10^ but its clinical value has not yet been realised, hindered partly by a lack of a clear understanding of how to quantify benefit or ensure patient safety, and increasing concerns about the ethical and medico-legal impact.^11^

This analysis is written with the dual aim of helping clinical safety professionals to critically appraise current medical AI research from a quality and safety perspective, and supporting research and development in AI by highlighting some of the clinical safety questions that must be considered if medical application of these exciting technologies is to be successful.

## Trends in ML research

Clinical decision support systems (DSS) are in widespread use in medicine and have had most impact providing guidance on the safe prescription of medicines,^12^ guideline adherence, simple risk screening^13^ or prognostic scoring.^14^ These systems use predefined rules, which have predictable behaviour and are usually shown to reduce clinical error,^12^ although sometimes inadvertently introduce safety issues themselves.^15 16^ Rules-based systems have also been developed to address diagnostic uncertainty^17–19^ but have struggled to deal with the breadth and variety of information involved in the typical diagnostic process, a problem for which ML systems are potentially better suited.

As a result of this gap, the bulk of research into medical applications of ML has focused on diagnostic decision support, often in a specific clinical domain such as radiology, using algorithms that learn to classify from training examples (supervised learning). Some of this research is beginning to be applied to clinical practice, and from these experiences lessons can be learnt about both quality and safety. Notable examples of this include the diagnosis of malignancy from photographs of skin lesions,^6^ prediction of sight-threatening eye disease from optical coherence tomography (OCT) scans^7^ and prediction of impending sepsis from a set of clinical observations and test results.^20 21^

Outside of diagnostic support ML systems are being developed to provide other kinds of decision support, such as providing risk predictions (eg, for sepsis^20^) based on a multitude of complex factors, or tailoring specific types of therapy to individuals. Systems are now entering clinical practice that can analyse CT scans of a patient with cancer and by combining this data with learning from previous patients, provide a radiation treatment recommendation, tailored to that patient which aims to minimise damage to nearby organs.^22^

Other earlier stage research in this area uses algorithms that learn strategies to maximise a ‘reward’ (reinforcement learning). These have been used to test approaches to other personalised treatment problems such as optimising a heparin loading regime to maximise time spent within the therapeutic range^23^ or targeting blood glucose control in septic patients to minimise mortality.^24^

Looking further ahead AI systems may develop that go beyond recommendation of clinical action. Such systems may, for example, autonomously triage patients or prioritise individual’s access to clinical services by screening referrals. Such systems could entail significant ethical issues by perpetuating inequality,^25^ analogous to those seen in the automation of job applicant screening,^26^ of which it is said that ‘blind confidence in automated e-recruitment systems could have a high societal cost, jeopardizing the right of individuals to equal opportunities in the job market’. This is a complex discussion and beyond the remit of this article.

Outside of medicine, the cutting edge of AI research is focused on systems that behave autonomously and continuously evolve strategies to achieve their goal (active learning), for example, mastering the game of Go,^27^ trading in financial markets,^28^ controlling data centre cooling systems^29^ or autonomous driving.^30 31^ The safety issues of such actively learning autonomous systems have been discussed theoretically by Amodei *e* *t al* 32 and from this work we can identify potential issues in medical applications. Autonomous systems are long way off practical implementation in medicine, but one can imagine a future where ‘closed loop’ applications, such as subcutaneous insulin pumps driven by information from wearable sensors,^33^ or automated ventilator control driven by physiological monitoring data in intensive care,^34^ are directly controlled by AI algorithms.

These various applications of ML require different algorithms, of which there are a great many. Their performance is often very dependent on the precise composition of their training data and other parameters selected during training. Even controlling for these factors some algorithms will not produce identical decisions when trained in identical circumstances. This makes it difficult to reproduce research findings and will make it difficult to implement ‘off the shelf’ ML systems. It is notable in ML literature that there is not yet an agreed way to report findings or even compare the accuracy of ML systems.^35 36^

**Figure 1:**
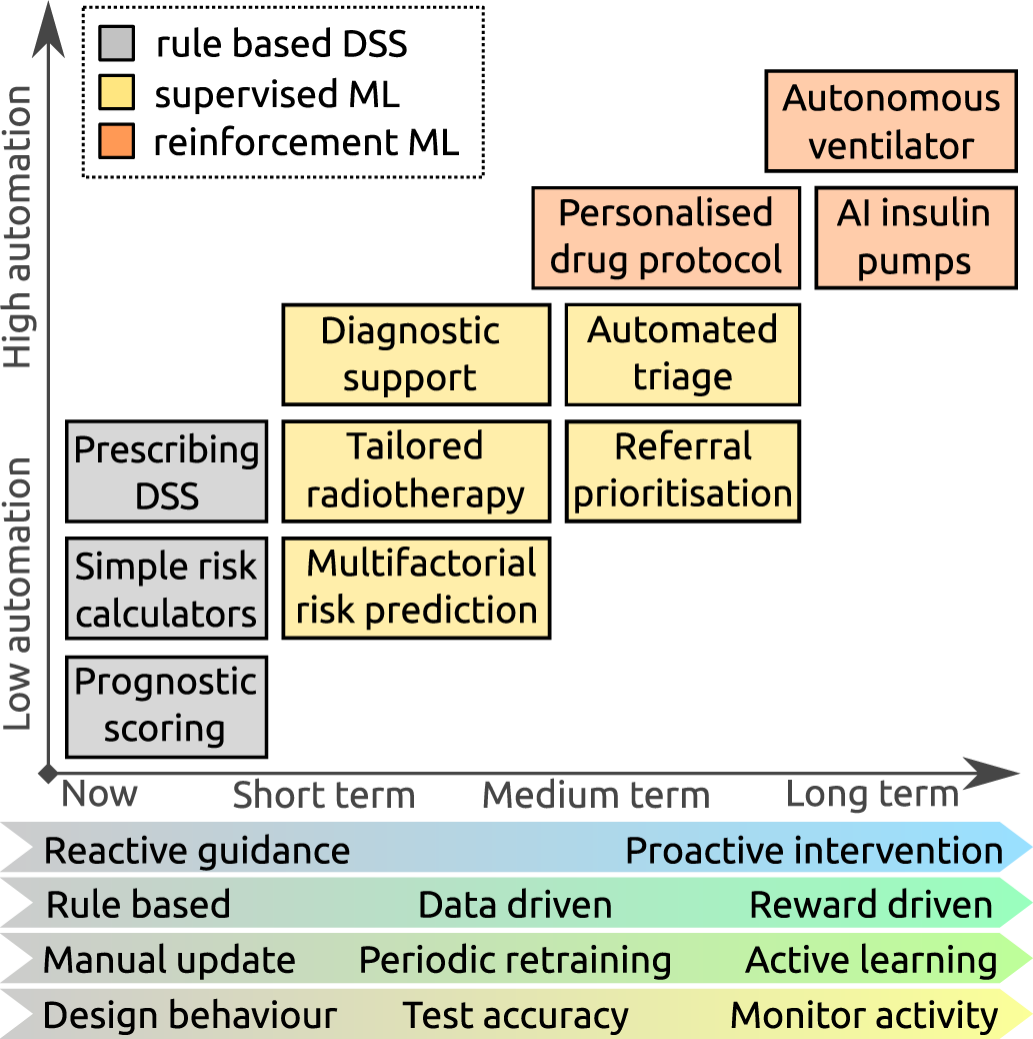
Expected trends in machine learning (ML) research: boxes show representative examples of decision support tasks that are currently offered by rule-based systems (grey), and hypothetical applications of ML systems in the future (yellow and orange), demonstrating increasing automation. The characteristics of the ML systems that support these tasks are anticipated to evolve, with systems becoming more proactive and reward driven, continuously learning to meet more complex applications, but potentially requiring more monitoring to ensure they are working as expected. AI, artificial intelligence; DSS, decision support systems.

Figure 1 summarises expected trends in ML research in medicine, over the short, medium and longer terms, with the focus evolving from reactive systems, trained to classify patients from gold standard cases, with a measurable degree of accuracy, to proactive autonomous systems which continuously learn from experience, whose performance is judged on outcome. Translation of ML research into clinical practice requires a robust demonstration that the systems function safely, and with this evolution different quality and safety issues present themselves.

## Quality and safety in ML systems

In an early AI experiment, the US army used ML to try to distinguish between images of armoured vehicles hidden in trees versus empty forests.^1^ After initial success on one set of images, the system performed no better than chance on a second set. It was subsequently found that the positive training images had all been taken on a sunny day, whereas it had been cloudy in the control photographs—the machine had learnt to discriminate between images of sunny and cloudy days, rather than to find the vehicles. This is an example of an unwittingly introduced bias in the training set. The subsequent application of the resulting system to unbiased cases is one cause of a phenomenon called ‘distributional shift’.

### Short-term issues

#### Distributional shift

Distributional shift^32^ is familiar to many clinicians, who find previous experience inadequate for new situations, and have to operate, cautiously, outside of a ‘comfort zone’. ML systems can be poor at recognising a relevant change in context or data, and this results in the system confidently continuing to make erroneous predictions based on ‘out-of-sample’ inputs.^32^

A mismatch between training and operational data can be inadvertently introduced, most commonly, as above, by deficiencies in the training data, but also by inappropriate application of a trained ML system to an unanticipated patient context. Such situations can be described as ‘out-of-sample’ input, and the need to cater for many such edge cases is described as the ‘Frame problem’^25^ of AI.

The limited availability of high quality data for training, correctly labelled with the outcome of interest, is a recurrent issue in ML studies. For example, when data are available it may have been collected as ‘interesting cases’ and not representative of the normal, leading to a sample selection bias.^6^ In another example, the outcome may be poorly defined (eg, pneumonia) and variably assigned by experts, leading to a training set with poor reproducibility, and no ‘ground truth’ to learn associations.^9^

Inappropriate application of an ML system to a different context can be quite subtle. De Fauw *et al* 7 discovered their system worked well on scans from one OCT machine, but not another, necessitating a process to normalise the data coming from each machine, before a diagnostic prediction could be made. Similarly we anticipate that the system for diagnosing skin malignancy,^6^ which was trained on pictures of lesions biopsied in a clinic, may not perform as well when applied to the task of screening the general population where the appearance of lesions, and patient’s risk profile, is different.

In some cases, distributional shift is introduced deliberately. ML systems perform best when index cases and controls are approximately equal in the training set,^37^ and this is not common in medicine. Imbalanced data sets may be ‘rebalanced’ by under-sampling or over-sampling, and without correction the resulting system will tend to over-diagnose the rare case.^38^ Alternative approaches may ‘boost’ the significance of true positive or false negative cases depending on the application, which can lead, for example, to a model good for screening but poor for diagnosis.^39^

Over time disease patterns change, leading to a mismatch between training and operational data. The effect of this on ML models of acute kidney injury was studied by Davis *et al,* 40 who found that over time decreasing AKI incidence was associated with increasing false positives from their ML system, an example of prediction drift.

There are many different ML algorithms, and they perform differently under the challenge of distributional shift, and this ‘may lead to arbitrary and sometimes deleterious effects that are costly to diagnose and address’.^41^ It is notable however that the sepsis detection system mentioned above^20^ has been successfully tested in the different context of a community hospital^5^ despite being trained in intensive care, a potential distributional shift, and thus shows some capability of adaptation through ‘transfer learning’.^38 42^

#### Insensitivity to impact

In the comparison between ML systems and expert dermatologists performed by Esteva *et al,* 6 both humans and machines find it difficult to discriminate between benign and malignant melanocytic lesions, but humans ‘err on the side of caution’ and over-diagnose malignancy. The same pattern was not observed for relatively benign conditions. While this decreases a clinician’s apparent accuracy, this behaviour alteration in the face of a potentially serious outcome is critical for safety, and something that the ML system has to replicate. ML systems applied to clinical care should be trained not just with the end result (eg, malignant or benign), but also with the cost of both potential missed diagnoses (false negatives) and over-diagnosis (false positives).^43^ During learning ML systems assess and maximise their performance based on a measure of accuracy obtained on predictions made from training data. Often this accuracy measure does not take into account real-world impacts, and as a result the ML system can be optimised for the wrong task, and comparisons to clinician’s performance flawed.

#### Black box decision-making

One of the key differences between rule-based systems and the multitude of ML algorithms is the degree to which the resulting prediction can be explained in terms of its inputs. Some ML algorithms, particularly those based on artificial neural networks, make inscrutable predictions and for these algorithms it is harder to detect error or bias. This issue was demonstrated by the armoured vehicle detection system developed by the US army described above^1^ and has been most studied in ML systems relying on image analysis.^6 9^ To mitigate this, such systems can produce ‘saliency maps’ which identify the areas of, for example, the skin lesion^6^ or the chest X-rays,^9^ which most contributed to their prediction. However, outside of image analysis this inscrutability is harder to manage, and detection of bias in black box algorithms requires careful statistical analysis of the behaviour of the model in the face of changing inputs.^44 45^

#### Unsafe failure mode

The concept of confidence of prediction was mentioned in the context of distributional shift above. As with interpretability, not all ML algorithms produce estimates of confidence. If ML systems are opaque to interpretation, it becomes essential for the clinician to be aware whether the system believes its prediction is a sensible one. If the system’s confidence is low, best practice design would be to fail-safe^46^ and refuse to make a prediction either way. A similar fail-safe may be needed if the system has insufficient input information or detects an ‘out-of-sample’ situation as described above.^46^

### Medium-term issues

#### Automation complacency

As humans, clinicians are susceptible to a range of cognitive biases which influence their ability to make accurate decisions.^47^ Particularly relevant is ‘confirmation bias’ in which clinicians give excessive significance to evidence which supports their presumed diagnosis and ignore evidence which refutes it.^25^ Automation bias^48^ describes the phenomenon whereby clinicians accept the guidance of an automated system and cease searching for confirmatory evidence (eg, see Tsai *et al* 49), perhaps transferring responsibility for decision-making onto the machine—an effect reportedly strongest when a machine advises that a case is normal.^48^ Automation complacency is a related concept^48^ in which people using imperfect DSS are least likely to catch errors if they are using a system which has been generally reliable, they are loaded with multiple concurrent tasks and they are at the end of their shift.

Automation complacency can occur for any type of decision support, but may be potentiated when combined with other pitfalls of ML described above. For example, given the sensitivity to distributional shift described, the usually reliable ML system that encounters an out-of-sample input may not ‘fail safely’ but continue confidently to make an erroneous prediction of low malignancy risk and not be questioned by the busy clinician who then ceases to consider alternatives.

#### Reinforcement of outmoded practice and self-fulfilling predictions

In the medium term, we expect to see systems emerging from research that use ML to recommend the most appropriate clinical actions, for example, by identifying patients who might benefit most from a specific treatment or for whom further referral and investigation is warranted.^7^

Such recommendation decision support already exists, but in systems whose behaviour is determined by explicitly designed rules. The shift to a data-driven approach introduces a new risk in the situation of a sudden change in clinical practice that requires the DSS to change, for example, a drug safety alert. While the rule-based system can be manually updated, as ML is predicated on the availability of appropriate data, it has the potential to reinforce outmoded practice, and a radical change that invalidates historical practice is difficult to absorb, as there are no prior data to retrain the system with. The need to periodically retrain and evaluate performance in response to technological evolution, new knowledge and protocol changes in medicine requires costly updating of gold standard data sets.

On the other hand, a related potential problem could arise in ML systems that are very frequently updated, and particularly those that continuously learn. Suppose a system predicts a prognosis, this may in turn influence therapy in a way that reinforces the prognosis and lead to a positive feedback loop. In this scenario, there is a self-fulfilling prediction, which then may be further reinforced as the ML system learns.

### Longer-term issues

Table 1 incorporates Amodei *et al*’s framework for safety in AI,^32^ which deals with issues more specific to continuously learning, autonomous systems. For obvious reasons, such systems will be challenging to deploy in the context of medicine and so their safety issues are less immediate. Rather than repeating Amodei *et al*’s detailed analysis,^32^ we describe these issues using hypothetical scenarios based on the research into personalised heparin dosing mentioned above^23^:

**Table 1:** A general framework for considering clinical artificial intelligence (AI) quality and safety issues in medicine

Issue	Summary	Example
***Short term***		
Distributional shift	A mismatch between the data or environment the system is trained on and that used in operation, due to bias in the training set, change over time, or use of the system in a different population, may result in an erroneous ‘out of sample’ prediction.	The accuracy of a system which predicts impending acute kidney injury based on other health records data, became less accurate over time as disease patterns changed.^40^
Insensitivity to impact	A system makes predictions that fail to take into account the impact of false positive or false negative predictions within the clinical context of use.	An unsafe diagnostic system is trained to be maximally accurate by correctly diagnosing benign lesions at the expense of occasionally missing malignancy.^6^
Black box decision making	A system’s predictions are not open to inspection or interpretation and can only be judged as correct based on the final outcome.	A X-Ray analysis AI system could be inaccurate in certain scenarios because of a problem with training data, but as a black box this is not possible to predict and will only become apparent after prolonged use.^9^
Unsafe failure mode	A system produces a prediction when it has no confidence in the prediction accuracy, or when it has insufficient information to make the prediction.	An unsafe AI decision support system may predict a low risk of a disease when some relevant data is missing. Without any information about the prediction confidence, a clinician may not realise how untrustworthy this prediction is.^46^
***Medium term***		
Automation complacency	A system’s predictions are given more weight than they deserve as the system is seen as infallible or confirming initial assumptions.	The busy clinician ceases to consider alternatives when a usually predictable AI system agrees with their diagnosis.^48^
Reinforcement of outmoded practice	A system is trained on historical data which reinforces existing practice, and cannot adapt to new developments or sudden changes in policy	A drug is withdrawn due to safety concerns but the AI decision support system cannot adapt as it has no historical data on the alternative.
Self-fulfilling prediction	Implementation of a system indirectly reinforces the outcome it is designed to detect.	A system trained on outcome data, predicts that certain cancer patients have a poor prognosis. This results in them having palliative rather than curative treatment, reinforcing the learnt behaviour.
***Long term***		
Negative side effects	System learns to perform a narrow function that fails to take account of some wider context creating a dangerous unintended consequence.	An autonomous ventilator derives a ventilation strategy that successfully maintains short term oxygenation at the expense of long-term lung damage.^34^
Reward hacking	A proxy for the intended goal is used as a ‘reward’ and a continuously learning system finds an unexpected way to achieve the reward without fulfilling the intended goal.	An autonomous heparin infusion finds a way to control activated partial thromboplastin time (aPTT) at the time of measurement without achieving long-term control.^23^
Unsafe exploration	An actively learning system begins to learn new strategies by testing boundary conditions in an unsafe way.	A continuously learning autonomous heparin infusion starts using dangerously large bolus doses to achieve rapid aPTT control.^23^
Unscalable oversight	A system requires a degree of monitoring that becomes prohibitively time consuming to provide.	An autonomous subcutaneous insulin pump requires the patient to provide exhaustive detail of everything they have eaten before it can adjust the insulin regime.^33^

- Negative side effects: The target of maximising the time in the therapeutic window requires careful management of heparin infusions that delay administration of other medications
- Reward hacking: An automated system may find ways in which to ‘game’ the goals defined by the reward function. The heparin dosing system, for example, might stumble on a strategy of giving pulses of heparin, immediately before activated partial thromboplastin time (aPTT) measurement, giving good short-term control, but without achieving the intended goal of stable long-term control. This is known as ‘hacking the reward function’ or ‘wireheading’.^32^
- Unsafe exploration: As part of its continuous learning, the system may experiment with the dosing of heparin to try and improve its current behaviour. How do we set limits to prevent dangerous overdosing, and define what changes in strategy are safe for the system to ‘explore’^50^?
- Unscalable oversight: As the system is learning new strategies for heparin management for novel patient groups, the management strategies it proposes require inconveniently frequent and expensive aPTT measurement.

At present these issues are merely theoretical in medicine, but they have been observed in ML test environments^51^ and are increasingly becoming relevant in applications such as autonomous driving systems.^31^

## Conclusion

Developing AI in health through the application of ML is a fertile area of research, but the rapid pace of change, diversity of different techniques and multiplicity of tuning parameters make it difficult to get a clear picture of how accurate these systems might be in clinical practice or how reproducible they are in different clinical contexts. This is compounded by a lack of consensus about how ML studies should report potential bias, for which the authors believe the Standards for Reporting of Diagnostic Accuracy initiative^52^ could be a useful starting point. Researchers need also to consider how ML models, like scientific data sets, can be licensed and distributed to facilitate reproduction of research results in different settings.

As ML matures we suggest a set of short-term and medium-term clinical safety issues (see table 1) that need addressing to bring these systems from laboratory to bedside. This framework is supported by a set of quality control questions (Box 1) that are designed to help clinical safety professionals and those involved in developing ML systems to identify areas of concern. Detailed mitigation of these issues is a large topic that cannot be addressed here, but is discussed by Amodei *et al* 32 and Varshney *et al*.^46^

Box 1 **- ﻿Quality control questions for short-term and medium-term issues in machine learning**

**Distributional shift**

- Has the system been tested in diverse locations, underlying software architectures (such as electronic health records), and populations?
- How can we be sure the training data matches what we expect to see in real life and does not contain bias?
  - How can we be confident of the quality of the ‘labels’ the system is trained on?
  - Do the ‘labels’ represent a concrete outcome (‘ground truth’) or a clinical opinion?
  - How has imbalance in the training set been addressed?
  - Is the system applied to the same diagnostic context that it was trained in?
- How is the system going to be monitored and maintained over time to adjust for prediction drift?

**Insensitivity to impact**

- Does the system adjust its behaviour (‘err on the side of caution’) where there are high impact negative outcomes?
- Can the system identify ‘out of sample’ input and adjust its confidence accordingly?

**Black box decision-making, unsafe failure and automation complacency**

- Are the system’s predictions interpretable?
- Does it produce an estimate of confidence?
- How is the certainty of prediction communicated to clinicians to avoid automation bias?

**Reinforcement of outmoded practice and self-fulfilling predictions**

- How can it accommodate breaking changes to clinical practice?
- What aspects of existing clinical practice does this system reinforce?

Implementation of ML DSS in the short term is likely to focus on diagnostic decision support. ML diagnostic decision support should be assessed in the same manner and with the same rigour as the development of a new laboratory screening test. Wherever possible a direct comparison should be sought to existing decision support or risk scoring systems—ideally through a randomised controlled trial as exemplified by Shimabukuro *et al*.^42 53^

As with all clinical safety discussions we need to maintain a realistic perspective. Suboptimal decision-making will happen with or without ML support, and we must balance the potential for improvement against the risk of negative outcomes.
